# Effects of 16-Week Consumption of Caffeinated and Decaffeinated Instant Coffee on Glucose Metabolism in a Randomized Controlled Trial

**DOI:** 10.1155/2012/207426

**Published:** 2012-11-05

**Authors:** Keizo Ohnaka, Mizuko Ikeda, Takako Maki, Tomoko Okada, Takao Shimazoe, Masahiro Adachi, Masatoshi Nomura, Ryoichi Takayanagi, Suminori Kono

**Affiliations:** ^1^Department of Geriatric Medicine, Graduate School of Medical Sciences, Kyushu University, Fukuoka 812-8582, Japan; ^2^Department of Preventive Medicine, Graduate School of Medical Sciences, Kyushu University, Fukuoka 812-8582, Japan; ^3^Department of Clinical Pharmacology, Graduate School of Pharmaceutical Sciences, Kyushu University, Fukuoka 812-8582, Japan; ^4^Department of Medicine and Bioregulatory Science, Graduate School of Medical Sciences, Higashi-ku, Fukuoka 812-8582, Japan

## Abstract

*Objective*. Observational studies have shown a protective association between coffee consumption and type 2 diabetes mellitus whereas caffeine or caffeinated coffee acutely deteriorates glucose tolerance. We investigated the effects of chronic drinking of instant coffee on glucose and insulin concentrations during a 75 g oral glucose tolerance test. *Methods*. Overweight men with a mild-to-moderate elevation of fasting plasma glucose were randomly allocated to a 16-week intervention of consuming 5 cups of caffeinated (*n* = 17) or decaffeinated (*n* = 15) instant coffee per day or no coffee (*n* = 13). *Results*. The caffeinated coffee group showed statistically significant decreases in the 2-hour concentrations and the area under the curve of glucose while neither decaffeinated coffee nor coffee group showed such a change. Waist circumstance decreased in the caffeinated coffee group, increased in the decaffeinated coffee group, and did not change in the noncoffee group (*P* = 0.002). With adjustment for the change in waist circumference, caffeinated and decaffeinated coffee consumption were associated with a modest decrease in the postload glucose levels. *Conclusion*. Both caffeinated and decaffeinated coffee may be protective against deterioration of glucose tolerance.

## 1. Introduction

Observational studies have consistently shown a protective association between coffee consumption and type 2 diabetes mellitus [1, 2]. Both caffeinated and decaffeinated coffee seem to be associated with a decreased risk of type 2 diabetes mellitus [3, 4], although a prospective study suggested a stronger association with decaffeinated coffee [5]. On the other hand, it is well documented that the ingestion of caffeine or caffeinated coffee acutely deteriorates glucose tolerance, as assessed by postprandial plasma glucose concentrations [6–10]. Caffeine administration is also shown to decrease the insulin-mediated glucose disposal in the hyperinsulinemic-euglycemic glucose clamp procedure in humans [11, 12]. It is thus hypothesized that coffee compounds other than caffeine exert a beneficial effect on glucose metabolism. Chlorogenic acids are such bioactive compounds of a potent antioxidant property [13, 14]. Alternatively, habitual coffee consumption may be protective against type 2 diabetes mellitus by mechanisms other than postulated for the acute effect of caffeine.

Few trials have addressed the long-term effect of caffeine or caffeinated coffee on glucose metabolism. Participants in the intervention group in these studies consumed caffeine (400 mg/day) for one week [15]; filtered coffee (900 mL/day) or caffeine (870 mg/day) for 2 weeks [16]; filtered coffee (1 L/day) for 4 weeks [16]; caffeinated or decaffeinated instant coffee (5 cups/day) for 8 weeks [17]. In a one-arm trial [18], the subjects consumed 4 cups of filtered coffee per day for 1 month and 8 cups of filtered coffee per day for another month. None of these studies showed a clear effect on either fasting [15, 16] or postload [17, 18] glucose concentrations, while some suggested an elevation of fasting insulin concentrations after one-week use of caffeine [15] and 4-week consumption of filtered coffee [16]. In a randomized controlled trial reported here, we investigated the effects of drinking caffeinated and decaffeinated instant coffee (5 cups/day) for 16 weeks on glucose and insulin concentrations during a 75 g oral glucose tolerance test (OGTT). We also evaluated the effects of coffee consumption on plasma adiponectin concentrations and serum C-reactive protein (CRP), because coffee consumption was reported to be associated with these inflammation-related biological markers in some [19, 20], but not all [21], human studies.

## 2. Methods

### 2.1. Participants

Participants were men aged 40–64 years who had body mass index of 25–30 kg/m^2^ and fasting plasma glucose of 100–140 mg/dL in the past year (or 90–140 mg/dL at screening in the case of no measurement in the past year). Exclusion criteria were disease under mediation, life-limiting disease without medication, a prior history of gastrectomy, being unable to drink coffee, and daily use of coffee. Participants were recruited by means of articles in nationwide newspapers, advertisements in local newspapers and magazines, posters at workplaces, and personal contact. The trial was approved by the ethics committee of Kyushu University Graduate School of Medical Sciences, and each participant gave written informed consent.

During the period from March 2008 to April 2009, a total of 276 men contacted us about the trial. After a prescreening by telephone and a screening examination, a total of 49 men entered the study.

### 2.2. Protocol

Eligible consenting participants entered a 2-week run-in period, followed by a 16-week intervention period. The run-in period was employed for washout of caffeine and for ensuring tolerability to caffeine depletion. After completion of the run-in period, subjects were randomly allocated to one of the following three treatments of 16 weeks, that is, 5 cups of caffeinated instant coffee per day, 5 cups of decaffeinated instant coffee per day, and no coffee. During the run-in and intervention periods, subjects were not allowed to consume caffeine-contained beverages and foods (coffee, tea, cola, cocoa, chocolate, and caffeine-contained supplements) except supplied coffee. They were instructed to maintain the current diet and physical activity during the study period. Mineral water (Coca-Cola MINAQUA) of an amount corresponding to two 500-mL bottles per day was supplied to all the subjects during the run-in period and to those in the noncoffee group during the intervention period. Subjects in the coffee groups were supplied with caffeinated or decaffeinated instant coffee (NESCAFE GOLDBLEND) of the same lot number and with mineral water of one 500-mL bottle per day during the intervention period.

Subjects were invited to a study site at the medical campus of Kyushu University prior to the run-in period and at weeks 0, 8, and 16 of intervention for 75-g OGGT. Randomization was done by permuted block design using blocks of different sizes (multiples of three) with each block consisting of equal numbers of the three assignments. The block size was adjusted to the number of subjects entering the intervention at different points of time. Each participant drew one tip from among the bag containing chips numbered sequentially and was allocated to an intervention group specified for the sequential number. Remaining chips were retained in the bag to which a new block of chips were added so that the bag always contained at least one for each intervention group. Study participants received $30 at screening, $100 at 0 and 8 weeks each, and $200 at 16 weeks ($1 = 100  Japanese yen).

### 2.3. Coffee Preparation

Subjects were instructed to prepare one cup/glass of coffee using one spoonful of instant coffee (approximately 1.2–1.3 g). Either hot or ice coffee was permitted, but coffee was drunk without sugar, milk, or any other additives. A required amount of instant coffee and two spoons of the same size were supplied to each subject in the caffeinated or decaffeinated coffee group.

### 2.4. Measurements

Subjects attended the screening at 10:00 hours, and those without a recorded measurement of fasting plasma glucose were instructed to be fast at least for 10 hours. Height and body weight were measured with subjects wearing light cloths without shoes. On scheduled visits at weeks 0, 8, and 16 of intervention, participants were instructed to be fast from 21:00 hours on the previous day and to be present at the study site at 09:30 hour. Body weight, waist circumference, and blood pressure were measured. Waist circumference was measured at the horizontal plane of the umbilical level with subjects wearing an undergarment at the expiratory phase of usual abdominal respiration. A 75 g OGTT was started at 10:00 hours, and venous blood was drawn at 0, 30, 60, 90, and 120 min after the glucose challenge. Plasma and serum were separated after centrifugation within two hours. Plasma samples for glucose and insulin measurements were stored at −20°C until the assay within 2 working days. Serum samples for caffeine, CRP, and adiponectin measurements were stored at −80°C until the completion of the trial.

All laboratory measurements except for caffeine were carried out at an external laboratory (SRL, Tokyo). Plasma glucose and insulin concentrations were determined by hexokinase method and chemiluminescent enzyme immunoassay, respectively. Serum concentrations of high-sensitivity CRP were measured by using a latex-enhanced immunonephelometric assay on a BN II analyzer (Siemens Healthcare Diagnostics, Marburg, Germany) [22]. Total adiponectin concentrations were measured by the ELISA method [23], and high-molecular weight (HMW) adiponectin concentrations were assayed by the two-step sandwich ELISA method [24]. For one subject in the decaffeinated coffee group, blood sampling was missed at 90 min during the OGTT at 8 weeks, and the glucose and insulin concentrations were imputed by averaging the 60 min and 120 min values. One in the caffeinated coffee group had CRP below the detection limit (0.05 mg/L) at the baseline and 16 weeks, and a value of 0.05 mg/L was imputed. In addition, CRP was greater than 10 mg/L at 16 weeks for one in the noncoffee group, and the value was discarded because such a high value is generally indicative of acute inflammation.

As for saliva collection (see below), 3 cotton balls of 1 cm in diameter were placed in the sublingual and cheek pouch after mouth rinse with a cup of water, and approximately 1 mL of saliva was collected into a tube by pressing wet cotton balls in a 5 mL syringe. Saliva solution was stored at −80°C. Pretreatment of saliva samples was performed by the method described elsewhere [25]. Serum and salivary caffeine concentrations were determined by high-performance liquid chromatography at Kyushu University Graduate School of Pharmaceutical Sciences [26]. Standard caffeine solutions of 1, 2, 5, 10, 25, 50, and 100 *μ*M were used for calibration. Thus the detection limit of caffeine concentration was 1 *μ*M.

### 2.5. Compliance

On each visit to the study site, a self-administered questionnaire ascertained smoking, alcohol drinking, use of caffeine-containing foods and beverages, and drug use during the between-visit period. The questionnaires at 8 and 16 weeks of intervention inquired about any changes in diet and physical activity. Serum and salivary caffeine concentrations were determined at weeks 0, 8, and 16 of intervention. Subjects were visited at their homes or workplaces without appointment twice during the intervention period, that is, each in the first 8 weeks and second 8 weeks.

### 2.6. Statistical Analyses

Areas under the curve (AUC) for glucose and insulin during the OGTT were calculated using the trapezoidal method. Composite insulin sensitivity index, which represents the whole-body insulin disposal, was calculated by the method proposed by Matsuda and DeFronzo [27]. Homeostasis model assessment of insulin resistance (HOMA-IR) was calculated [28].

The distributions of laboratory measurements were mostly skewed to the right side, and median and interquartile range (IQR) was used to present the measurements at baseline. Analysis of variance, *χ* ^2^-test, and Kruskal-Wallis test were used for the three-group comparisons of means, proportions, and medians, respectively. As for the changes in laboratory measurements after the intervention, we used mean and standard deviation (SD) of the change for ease of presentation with nonparametric methods for statistical tests (Kruskal-Wallis test and Wilcoxon rank-sum test for the between-group comparison and Wilcoxon singed-rank test for the within-group comparison). We also performed the analysis by using the values at different points of time transformed to natural logarithms and assessed the mean and 95% confidence interval (CI) of the percent change from the baseline by analysis of variance. Exponentiation of the mean difference in the log-scale necessarily corresponds to the average percent change from the baseline. Pearson correlation coefficients were calculated to evaluate how changes in body weight and waist circumference were correlated with changes in glucose metabolism parameters. Analysis of covariance was used to obtain adjusted mean percent changes in glucose metabolism parameters with control for change in waist circumference and to assess the overall difference among the three groups. Linear regression coefficients for the indicator variables corresponding to the treatment categories (caffeinated and decaffeinated coffee) were used for the between-group difference as compared with the noncoffee group. Statistical significance was declared if two-sided *P* value was less than 0.05. All statistical analyses were performed by Stata Statistical Software version 10 (Stata Corporation, College Station, TX).

## 3. Results

### 3.1. Baseline Characteristics

**Figure 1:**
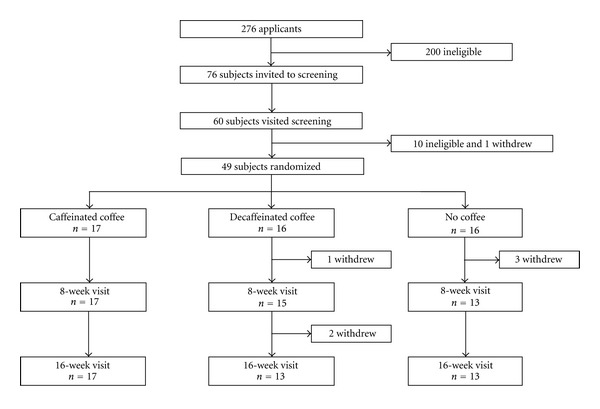
Trial profile.

As shown in Figure 1, a total of 49 men were randomly allocated to one of the three groups: caffeinated coffee (*n* = 17), decaffeinated coffee (*n* = 16), and noncoffee (*n* = 16). After the baseline OGTT, 4 men withdrew from the trial for diabetes mellitus requiring treatment (decaffeinated coffee group, *n* = 1; noncoffee group, *n* = 1) and hospitalization for acute diseases (noncoffee group, *n* = 2). Furthermore, after the 8-week OGTT, 2 men withdrew for diabetes mellitus requiring treatment (decaffeinated coffee group, *n* = 1) and treatment of hypertension (decaffeinated coffee group, *n* = 1). Withdrawal of the 3 men for treatment of diabetes mellitus was decided on the basis of the OGTT in the study. Thus the study subjects were 45 men with the 8-week measurements, and the analysis on the changes at 16 weeks was confined to 43 men with the 16-week measurements.

Age of the subjects ranged from 40 to 64 years with a mean of 52.7 years (SD 7.9 years). Smokers numbered 11 (24.4%), and median amounts of coffee and tea (green, black, and oolong tea combined) consumption were 2 cups per week (IQR 0.5–4.0) and 5.5 cups per week (IQR 2–21), respectively. There was no appreciable difference among the three intervention groups with respect to age (*P* = 0.46), smoking (*P* = 0.69), coffee use (*P* = 0.55), and tea consumption (*P* = 0.69).

None of the glucose and insulin parameters showed a measurable variation among the three groups (Table 1). Adiponectin and CRP concentrations were also similar in the three groups.

### 3.2. Changes during the Intervention

Mean changes of the laboratory parameters at 8 weeks and 16 weeks of the treatment are summarized in Tables 2 and 3, respectively. The caffeinated coffee group showed statistically significant decreases in the 2-hour glucose and AUC glucose at 16 weeks (Table 3), but not at 8 weeks (Table 2), as compared with the baseline values. Neither decaffeinated coffee nor noncoffee group showed such decreases. These decrease at 16 weeks among the caffeinated coffee group also statistically significantly differed from the changes observed in the noncoffee group. The average percent decreases were 13.1% (95% CI 1.6–23.2) for 2-hour glucose and 7.5% (95% CI 1.1–13.5) for AUC glucose after a 16-week consumption of caffeinated coffee. Insulin parameters including the composite ISI and HOMA-IR did not change materially during the intervention in any treatment groups and showed no between-group difference in the change.

Although the nonparametric analysis showed no statistically significant change in total or HMW adiponectin in any treatment groups (Tables 2 and 3), the analysis using the mean percent change showed that total adiponectin at 8 and 16 weeks and HMW adiponectin at 16 weeks increased statistically significantly, as compared with the baseline, in the caffeinated coffee group while these increases did not differ from the changes observed in the noncoffee group. The mean percent increases of total adiponectin were 6.0% (95% CI 0.2–12.0) at 8 weeks and 8.9% (95% CI 1.8–16.4) at 16 weeks, and the mean percent increase of HMW adiponectin at 16 weeks was 13.2% (95% CI 0.8–27.2).

Body weight and waist circumference did not change in any of the three groups after 8 weeks of intervention (data not shown). At 16 weeks, however, waist circumference decreased by 1.5 cm (95% CI 0.6–2.5) in the caffeinated coffee group and increased by 1.3 cm (95% CI 0.2–2.4) in the decaffeinated coffee group while a small decrease of 0.6 cm (95% CI −0.5 to 1.7) was observed in the noncoffee group (overall *P* = 0.002). Body weight at 16 weeks also showed a similar, but less prominent, pattern; the changes from the baseline were −1.1 kg (95% CI −2.0 to −0.1) in the caffeinated coffee group, 0.5 kg (95% CI −0.6 to 1.6) in the decaffeinated coffee group, and −0.6 kg (95% CI −1.7 to 0.5) in the noncoffee group (overall *P* = 0.10). The 16-week change in waist circumference was fairly strongly correlated with the changes in the log-scale of the 2-hour glucose (correlation coefficient *r* = 0.403) and AUC glucose (*r* = 0.399), but the correlation coefficients for the other parameters were relatively small: fasting glucose 0.06, fasting insulin 0.13, 2-hour insulin 0.19, AUC insulin 0.14, ISI −0.21, HOMA-IR 0.12, total adiponectin −0.21, HMW adiponectin −0.13, and CRP 0.17.

When statistical adjustment was made for the 16-week change in waist circumference, the percent changes in 2-hour and AUC values of glucose concentrations were attenuated in the caffeinated coffee group and were accentuated in the decaffeinated coffee group. The adjusted mean percent changes of 2-hour glucose at 16 weeks were −8.2% (95% CI −18.8 to 3.9) in the caffeinated coffee group, −7.7% (95% CI −20.4 to 6.9) in the decaffeinated coffee group, and 9.0% (95% CI −4.5 to 24.5) in the noncoffee group (overall *P* = 0.11); a pooled average percent decrease in the caffeinated and decaffeinated coffee group was significantly different as compared with the noncoffee group (*P* = 0.04). The adjusted mean percent changes of AUC glucose were −4.5% (95% CI −10.5 to 2.0) for caffeinated coffee, −7.6% (95% CI −14.6 to −0.1) for decaffeinated coffee, and 2.2% (95% CI −4.8 to 9.7) for control (overall *P* = 0.14); a pooled percent decrease in the two coffee groups was nearly significant as compared with the noncoffee group (*P* = 0.053).

### 3.3. Compliance

**Figure 2:**
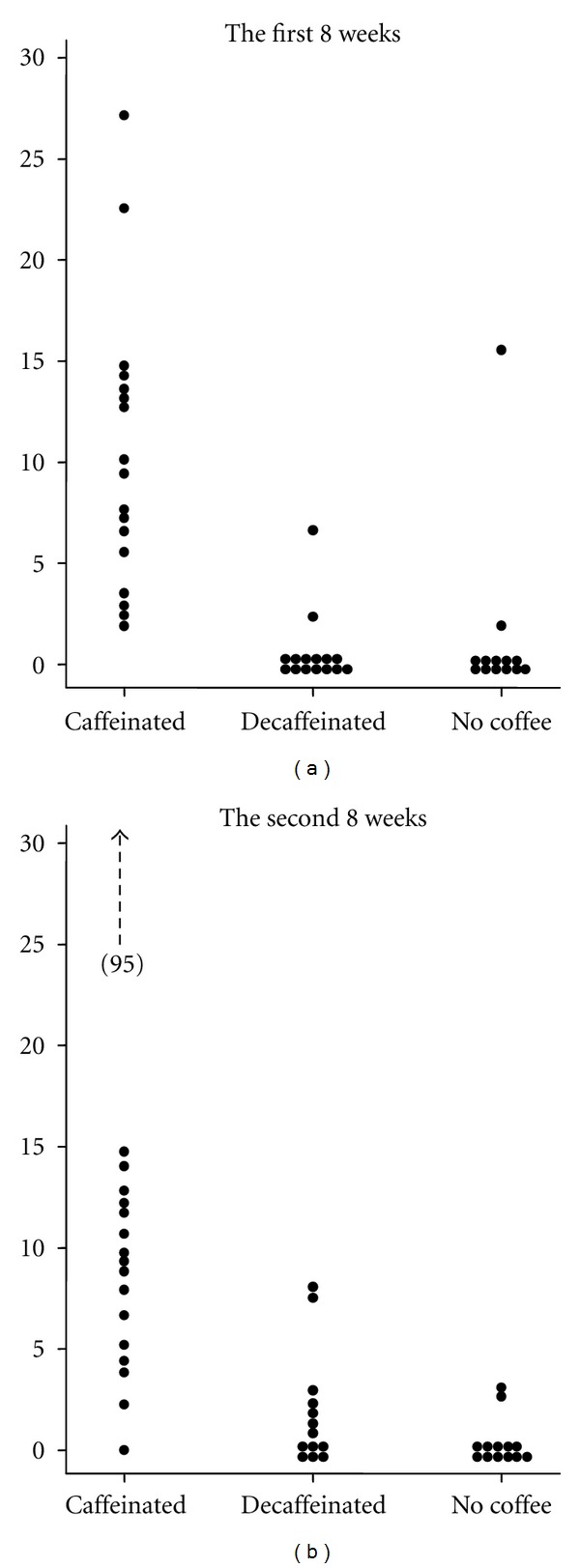
Caffeine concentrations (*μ*M) in saliva collected at unannounced visits in the first 8 weeks (a) and the second 8 weeks (b) during the intervention.

 At the baseline after the 2-week abstinence from caffeine, 36 (80%) of the 45 men were negative for serum caffeine; 8 (18.6%) had serum caffeine concentrations of 1.2–3.3 *μ*M, and one (2.3%) had 10.3 *μ*M of serum caffeine. Caffeine concentrations in saliva collected by unannounced visits are shown in Figure 2. Median concentrations of salivary caffeine in the caffeinated coffee group were 9.5 *μ*M (IQR 5.6–13.4 *μ*M) during the first 8 weeks and 9.3 *μ*M (IQR 5.2–12.1 *μ*M) during the second 8 weeks. Few men in the decaffeinated coffee group (*n* = 3) and noncoffee group (*n* = 1) had salivary caffeine concentrations of >5 *μ*M at either occasion.

In the caffeinated coffee group, median concentrations of serum caffeine were 6.9 *μ*M (IQR 3.2–9.9 *μ*M) at the 8-week visit and 8.2 *μ*M (IQR 5.3–12.4 *μ*M) at the 16-week visit. High concentrations of serum caffeine (>5 *μ*M) were detected for 3 subjects in the decaffeinated coffee group and for one subject in the noncoffee group at either of the scheduled visits during the intervention period.

Two men in the caffeinated coffee group and 3 men in the decaffeinated coffee group reported changes in physical activity at weeks 8 or 16. In the former group, one reported a lowered physical activity and the other reported an increase in physical activity. All of the 3 men in the decaffeinated coffee group reported a decrease in physical activity. The exclusion of these 5 men slightly attenuated the between-group difference in the change of waist circumference; mean changes at 16 weeks were −1.3 cm (95% CI −2.3 to −0.2 cm) in the caffeinated coffee group; 0.9 cm (95% CI −0.4 to 2.2 cm) in the decaffeinated coffee group; −0.6 cm (95% CI −1.7 to 0.5 cm) in the noncoffee group (overall *P* = 0.04).

## 4. Discussion

Modest decreases in 2-hour glucose and AUC glucose were observed after a 16-week consumption of 5 cups of caffeinated instant coffee per day. A consumption of decaffeinated coffee did not show such decreases. However, waist circumference decreased in the caffeinated coffee group and increased in the decaffeinated coffee group. With allowance for the change in waist circumference, the postload glucose levels seemed to be lowered after a 16-week consumption of caffeinated or decaffeinated coffee.

The differential change in waist circumstance between the caffeinated and decaffeinated groups was indeed problematic. An apparent increase in waist circumference after a 16-week consumption of decaffeinated coffee was ascribed partly to a decrease in physical activity as reported by 3 men. In the caffeinated coffee group, physical activity did not reportedly change to such an extent, but slight decreases in body weight and waist circumference may have been due to an unreported change in diet or physical activity. The decreases in body weight and waist circumference in the caffeinated coffee group may be regarded as compatible with caffeine's effects of increasing thermogenesis and fat oxidation [29]. In a meta-analysis of chamber studies on humans [30], a dose of 300 mg/day of caffeine was associated with an 80 kcal increase of energy expenditure per day. Caffeine enhances the release of epinephrine and free fatty acids in a fasting condition [6–10], and these physiological effects may be linked to the increase in energy expenditure [29]. However, it remains uncertain whether a long-term use of caffeine is beneficial in maintaining body weight or decreasing body fat, while an increase in caffeine intake was reported to be associated with a small reduction in long-term weight in an observational study [31].

The present study suggested that both caffeinated and decaffeinated coffee were associated with a modest improvement in the 2-hour glucose concentrations. The present findings are consistent with the results from observational studies [32, 33]. These studies consistently showed that coffee consumption was more strongly associated with lower concentrations of 2-hour glucose than of fasting glucose during a 75 g OGTT [32, 33]. The present study adds to evidence that coffee compounds other than caffeine are protective in glucose metabolism. Chlorogenic acids and other noncaffeine coffee compounds may exert protective effects by decreasing hepatic glucose production through inhibition of hepatic glucose-6-phosphate translocase [34], delaying intestinal glucose absorption as suggested by an altered profile of plasma concentrations of gastrointestinal hormones [35], and increasing whole-body glucose disposal or insulin sensitivity [36]. Coffee polyphenols were also shown to be protective against the damage of pancreatic islet caused by oxidative stress [37].

There is no doubt that caffeine or caffeinated coffee deteriorates glucose tolerance when administered prior to glucose load or meal. This adverse effect was observed not only in healthy subjects [6, 10] but also in patients with type 2 diabetes mellitus [7, 38, 39], with a habitual consumption of caffeinated coffee of different types. The caffeine's acute effect on glucose tolerance does not seem to diminish or weaken with a habitual consumption of caffeinated coffee [38]. Further studies are needed to elucidate a mechanism or mechanisms for a possible protective effect of a habitual use of caffeinated coffee as well as of decaffeinated coffee in glucose metabolism.

The insulin parameters did not change by either caffeinated or decaffeinated coffee in the present study. The changes in the insulin-related parameters generally showed a large between-subject variation, and it may have been difficult to detect a possible effect of coffee on the insulin parameters. A suggestive increase in adiponectin concentrations observed for the caffeinated coffee group is consistent with the previous observation [17], but the increase did not differ from the change in the noncoffee group.

There was a fairly large variation in serum and salivary caffeine concentrations in the caffeinated coffee group. Caffeine is metabolized almost exclusively by CYP1A2 in the liver. Functional genetic polymorphisms are known in the CYP1A2 gene, and the between-subject variation in caffeine metabolism is well known [40]. Thus low caffeine concentrations do not necessarily indicate poor compliance in the caffeinated coffee group.

The 16-week intervention period, use of the standard test for glucose tolerance, and saliva collection without appointment were advantages in the present studies. However, there were several limitations. We did not have direct information as to how stable the dietary intake and physical activity were during the intervention period. Statistical adjustment was made for the change in waist circumstance, but this measure alone probably did not capture the changes in physical activity and diet which would have affected glucose metabolism. The treatment was not blind to either the participants or the investigators, but this lack of blindness did not affect the laboratory measurements. Coffee was consumed without any additives in the present study, and the findings may not be applicable to coffee drinking with sugar and/or milk. Finally, we did not measure chemical compounds contained in caffeinated and decaffeinated coffee. It was previously reported that decaffeinated instant coffee contained a slightly lower amount of chlorogenic acids than caffeinated instant coffee of the same brand [17].

## 5. Conclusion

In overweight men with a mild-to-moderate elevation of fasting glucose concentrations, the 2-hour glucose and AUC glucose during a 75 g OGTT decreased after a 16-week consumption of 5 cups of caffeinated instant coffee per day. However, with adjustment for the change in waist circumference, both caffeinated and decaffeinated coffee seemed to be associated with lowered levels of the postload glucose. Habitual use of both caffeinated and decaffeinated coffee may be protective against deterioration of glucose tolerance.

## Figures and Tables

**Table 1:** Anthropometric measures, glucose metabolism parameters, and serum adiponectin and C-reactive protein at baseline.

Parameter (unit)	Caffeinated coffee (*n* = 17)	Decaffeinated coffee (*n* = 15)	No coffee (*n* = 13)	*P* value*
Body weight (kg)	77 (70–84)	75 (71–80)	79 (78–83)	0.22
Waist circumference (cm)	93 (89–99)	91 (88–95)	95 (90–97)	0.46
Plasma glucose (mmol/L)				
Fasting	6.0 (5.3–6.2)	6.1 (5.3–7.4)	5.8 (5.4–6.3)	0.68
2-hour	8.5 (7.3–9.9)	10.1 (7.7–12.8)	9.2 (7.4–12.9)	0.35
AUC^†^	18.3 (15.9–20.1)	20.6 (17.5–23.7)	20.8 (18.5–23.2)	0.16
Plasma insulin (pmol/L)				
Fasting	57 (38–66)	34 (27–71)	58 (52–66)	0.53
2-hour	551 (347–868)	314 (187–565)	472 (312–854)	0.24
AUC^†^	999 (676–1289)	570 (376–978)	881 (629–1057)	0.13
Composite ISI	3.6 (2.3–4.6)	4.8 (2.4–7.9)	3.4 (2.1–5.0)	0.38
HOMA-IR	2.0 (1.3–2.5)	1.2 (0.9–3.3)	2.0 (1.8–2.9)	0.51
Total adiponectin (*μ*g/mL)	4.9 (4.1–8.2)	5.8 (5.6–6.3)	4.8 (3.9–6.5)	0.66
HMW adiponectin (*μ*g/mL)	2.6 (1.5–4.9)	3.1 (2.2–4.4)	2.2 (1.7–3.6)	0.73
C-reactive protein (mg/L)	0.49 (0.26–0.85)	0.23 (0.19–0.83)	0.42 (0.29–0.72)	0.24

AUC: area under the curve; HMW: high-molecular weight; HOMA-IR: homeostasis model assessment of insulin resistance; ISI: insulin sensitivity index.

Values are medians and interquartile ranges (25th and 75th percentiles) in parentheses.

*Overall difference based on Kruskal-Wallis test.

^†^Hour was used for the time scale in the oral glucose tolerance test.

**Table 2:** Mean changes of glucose metabolism parameters and serum adiponectin and C-reactive protein at 8 weeks of intervention as compared with the baseline values.

Parameter	Caffeinated coffee (*n* = 17)	Decaffeinated coffee (*n* = 15)	No coffee (*n* = 13)	*P* value*
Plasma glucose (mmol/L)				
Fasting	0.0 (0.4)	0.1 (0.4)	−0.0 (0.2)	0.55
2-hour	−1.1 (2.4)	−0.5 (2.2)	−0.3 (1.6)	0.51
AUC^†^	−0.7 (2.3)	−0.3 (1.8)	−0.6 (1.2)	0.62
Plasma insulin (pmol/L)				
Fasting	3 (21)	5 (19)	−7 (24)	0.53
2-hour	−44 (315)	−6 (192)	18 (319)	0.99
AUC^†^	7 (234)	−5 (291)	−12 (214)	0.82
Composite ISI	−0.2 (1.5)	−0.1 (1.9)	0.2 (0.8)	0.46
HOMA-IR	0.1 (0.9)	0.2 (0.8)	−0.3 (1.0)	0.27
Total adiponectin (*μ*g/mL)^‡^	0.2 (0.9)	−0.1 (0.5)	−0.0 (0.7)	0.15
HMW adiponectin (*μ*g/mL)^‡^	0.1 (0.6)	−0.1 (0.6)	0.0 (0.7)	0.48
C-reactive protein (mg/L)^†^	−0.39 (1.54)	0.18 (0.62)	−0.12 (0.57)	0.80

AUC: area under the curve; HMW: high-molecular weight; HOMA-IR: homeostasis model assessment of insulin resistance; ISI: insulin sensitivity index.

Values in parentheses are standard deviations.

*Overall difference based on Kruskal-Wallis test.

^†^Hour was used for the time scale in the oral glucose tolerance test.

^‡^Number of the subjects in the decaffeinated coffee group was 13.

**Table 3:** Mean changes of glucose metabolism parameters and serum adiponectin and C-reactive protein at 16 weeks of intervention as compared with the baseline values.

Parameter	Caffeinated coffee (*n* = 17)	Decaffeinated coffee (*n* = 13)	No coffee (*n* = 13)	*P* value*
Plasma glucose (mmol/L)				
Fasting	−0.0 (0.5)	0.0 (0.7)	−0.0 (0.5)	0.88
2-hour	−1.3 (2.2)^†^	0.1 (2.6)	0.7 (1.4)	0.07
AUC^‡^	−1.4 (2.6)^†^	−0.3 (3.3)	0.3 (1.6)	0.08
Plasma insulin (pmol/L)				
Fasting	4 (23)	5 (20)	5 (16)	0.94
2-hour	−2 (515)	102 (896)	90 (260)	0.40
AUC^‡^	115 (526)	63 (771)	36 (252)	0.94
Composite ISI	−0.2 (1.7)	−0.4 (1.5)	−0.3 (1.0)	0.85
HOMA-IR	0.1 (0.9)	0.2 (0.8)	0.1 (0.7)	0.86
Total adiponectin (*μ*g/mL)	0.4 (0.9)	0.0 (0.5)	0.4 (1.1)	0.45
HMW adiponectin (*μ*g/mL)	0.3 (0.8)	−0.0 (0.5)	0.3 (0.7)	0.40
C-reactive protein (mg/L)	−0.43 (1.33)	0.42 (1.16)	−0.21 (0.66)	0.63

AUC: area under the curve; HMW: high-molecular weight; HOMA-IR: homeostasis model assessment of insulin resistance; ISI: insulin sensitivity index.

Values in parentheses are standard deviations.

*Overall difference based on Kruskal-Wallis test.

^†^ *P* < 0.05 as compared with the baseline and as compared with the noncoffee group.

^‡^Hour was used for the time scale in the oral glucose tolerance test.
